# A new technique for minimal invasive complete spinal cord injury in minipigs

**DOI:** 10.1007/s00701-017-3442-3

**Published:** 2018-01-12

**Authors:** Elena E. Foditsch, Gratian Miclaus, Irina Patras, Ioan Hutu, Karin Roider, Sophina Bauer, Günter Janetschek, Ludwig Aigner, Reinhold Zimmermann

**Affiliations:** 1 Spinal Cord Injury and Tissue Regeneration Center Salzburg, Paracelsus Medical University, Strubergasse 21, 5020 Salzburg, Austria; 2 SCM Neuromed, Timisoara, Romania; 3 Banat University of Agricultural Sciences and Veterinary Medicine, Timisoara, Romania; 4 University Clinics of Urology and Andrology, General Hospital Salzburg, Paracelsus Medical University, Salzburg, Austria; 5 Institute of Molecular Regenerative Medicine, Spinal Cord Injury and Tissue Regeneration Center Salzburg, Paracelsus Medical University, Salzburg, Austria; 6 American Hospital Dubai, Dubai, United Arab Emirates

**Keywords:** Spinal cord injury, Minipig, Minimal invasive, Computed tomography, Recovery

## Abstract

**Background:**

The aim of this study was to develop a minimal invasive complete spinal cord injury (SCI) minipig model for future research applications. The minipig is considered a translationally relevant model for SCI research. However, a standardized minimal invasive complete SCI model for pigs has not yet been established.

**Methods:**

Adult Göttingen minipigs were anesthetized and placed in extended prone position. After initial computed tomography (CT) scan, the skin was incised, a needle placed in the epidural fatty tissue. Using the Seldinger technique, a guidewire and dilators were introduced to insert the balloon catheter to Th12. After confirmation of the level Th11/Th12, the balloon was inflated to 2 atm for 30 min. The severity of the lesion was followed by CT and by MRI, and by immunohistochemistry. Function was assessed at the motor and sensory level.

**Results:**

Duration of procedure was about 60 min including the 30-min compression time. The balloon pressure of 2 atm was maintained without losses. The lesion site was clearly discernible and no intradural bleeding was observed by CT. Neurological assessments during the 4-month follow-up time showed consistent, predictable, and stable neurological deficits. Magnetic resonance imaging analyses at 6 h and 4 weeks post SCI with final immunohistochemical analyses of spinal cord tissue underlined the neurological outcomes and proved SCI completeness.

**Conclusions:**

We have established a new, minimal invasive, highly standardized, CT-guided spinal cord injury procedure for minipigs. All risks of the open surgery can be excluded using this technique. This CT-guided SC compression is an excellent technique as it avoids long surgery and extensive trauma and allows a feasible inter-animal comparison.

## Introduction

Traumatic spinal cord injury (tSCI) is a devastating condition with significant immediate loss of motor and sensory functions and subsequent morbidities and complications such as autonomic nervous system disturbances, loss of bladder and bowel control with increased risk for upper urinary tract infections, loss of sexual function, development of decubitus, and ulcers, having an irreversible impact on quality of life [1, 2]. Currently, after stabilization of the spine, the only treatment for SCI patients is rehabilitation with the aim to regain some functions and to reintegrate patients into a routine and social life [3]. tSCIs in humans are mainly contusions caused for example by traffic or sport accidents or by falls, resulting in dislocation of vertebral columns after an initial traumatic event [4, 5].

The majority of tSCI animal experiments are performed in rodents, in particular in mice and rats [6, 7]. Even though huge progress has been made in the preclinical field in the understanding of the tSCI pathophysiology, in the identification of therapeutically relevant targets, and in the development of molecular, cellular, and technology-supported therapies, none of these approaches has reached approval as a standard therapy [8]. Among many reasons for this are (i) general low translational value of rodent models, (ii) different neuroanatomical features of rodent and human spinal cord such as different locations of the corticospinal tracts, (iii) the usage of SCI lesion models with little relevance for human SCI, for example, the wire knife transection model [6, 8, 9].

An animal model that offers several advantages for the development of SCI therapies and their clinical translation is the minipig [10–12]. In contrast to rodents, but similar to other large animal models such as dogs, cats, sheep, and of course primates, the minipig has an anatomy, physiology, and pathophysiology relatively similar to humans. With respect to the central nervous system, this includes, for example, (i) the anatomical position of the corticospinal tract (CST), (ii) the gray and white matter distribution in general, (iii) the anatomy of spinal blood vessels and the blood flow characteristics, (iv) brain and spinal cord growth and development, and (v) the fact that the porcine and human spinal cord, but not that of rodents, are environed by cerebrospinal fluid (CSF) [7, 13, 14]. In addition, surgical and analytical instrumentations for interventions, as well as therapeutic devices such as electrodes and pacemakers in large animals such as minipigs are similar or even equal to the ones used in humans. Of particular interest for preclinical research and development is the Göttingen minipig strain, due to its gentle disposition and good training success rates, and due to the tolerance of even severe neurological deficits [15]. In the present study, we describe a new, minimal invasive, computed tomography (CT)-guided and controlled balloon compression technique for Göttingen minipigs. The primary goal was to create a new minimal invasive, CT-guided, highly standardized, technique to produce a complete tSCI in minipigs as a model for future translational studies.

## Methods and materials

### Animals

This study was carried out under the protocols approved by the Ethics and Deontology Committee for Research on Animals, Victor Babes University of Medicine, Timisoara, Romania. We certify that all applicable institutional and governmental regulations concerning the ethical use of animals were followed during the course of this research. The study was performed to minimize group size and animal suffering. Ten female Göttingen minipigs (Ellegaard Minipigs, Dalmose, Denmark) aged 6 months (weight 21–26 kg) were included in this study. The animals were group housed in the “Horia Cernescu” Animal Facility at the University of Agronomy and Veterinary Medicine in Temeshaw, Romania. All animals were housed in stable groups of three animals per pen and had 2 months of acclimatization. Prior to the SCI procedure, the health state of all animals was documented.

### Animal surgery

Minipigs were premedicated with an intramuscular cocktail of ketamine (15 mg/kg) and xylazine (2 mg/kg). Deep sedo-analgesia was established and maintained by intravenous propofol (10 mg/kg). Fentanyl (0.05 mg/kg) was given as analgesia prior to the surgery and a second time prior to the spinal cord compression. We preferred sedo-analgesia to general anesthesia with automated ventilation similar to the process of needle and balloon catheter placement. Prior to inflation of the balloon catheter, fentanyl was again given intravenously, to reduce possible pain perception. The observed muscular reaction during balloon catheter inflation was also observed when severing the SC during SC transection in deeply generally anesthetized pigs. After induction of anesthesia, the animals were placed in extended prone position. An initial CT image (Siemens, Somatom Sensation, Bucarest, Romania) of the spinal cord was performed, and the dimensions of the spinal cord were measured. The depth and angle for needle guidance to the intervertebral thoracic space T13/L1 were calculated. Needle positioning and individual virtual piercing routes were marked on the skin. After a minimal skin incision, the spinal canal (SC) was punctured by a 2-part 17.5-G puncture needle (Renodrain Nephrostomy Puncture Needle, Urotech, Achenmühle, Germany) under CT guidance. The needle was introduced into the epidural fatty tissue without piercing the underlying dura mater. Cerebrospinal fluid leakage was checked by aspiration testing. By means of the Seldinger technique, a hydrophilic guide wire (.035 × 150 cm, NiCore guidewire, Bard Medical, Covington, USA) was dorsally inserted via the puncture needle to the epidural space until thoracic spinal cord level Th8. The needle was removed and a 10-French dilator, followed by a 12-French dilator, was inserted via the guidewire into the epidural space to widen the channel. Thereafter, a kyphoplasty balloon catheter (Guardian Inflatable Bone Expander System, Taipeh, Taiwan, Balloon catheter 2ea) with radio-opaque markers was inserted via guide wire to the thoracic spinal cord level T12. All steps were carefully followed by CT scans. Once the position was confirmed by CT, the balloon was inflated to 2 atm. Balloon filling and spinal cord compression was confirmed in the CT and maintained for 30 min. The pressure was constantly monitored by a manometer to ensure continuous and constant pressure. After completion, the balloon was deflated and the balloon catheter including the guide wire was removed. The skin incision did not require a suture.

### Animal follow-up procedures

Animals were provided with antibiotics (ceftazidimim 40 mg/kg) and analgesics (butomidor 0.1 mg/kg) for 5 consecutive days post injury after standard protocols. A daily health examination of the injury site and skin analysis was performed. After 4 and 16 weeks, neurological assessments were conducted. Measurement of motor function, sensory function and muscle hypertonia was performed after standard protocols [7].

### Functional assessments

Motor function: Recovery of motor function was assessed using the 14-point scoring system (porcine neurological motor score—PNM score). Using this system, (i) movement in all three joints in the lower extremities and tail, with no weight support and (ii) the degree of recovery of ambulatory function, with weight support could be assessed [7]. Testing of motor function was performed after 4 and 16 weeks of follow-up time in the open field and on a treadmill by two experienced investigators including a video assessment to define the ultimate PMC scoring.

Sensory function: Sensory function was assessed by the presence of the withdrawal response to a mechanical stimulus. For this purpose, the minipigs were placed in a hammock. The toes of the front and hind limbs were progressively compressed with Halsted forceps. If sensation was present, a vigorous withdrawal response and/or vocalization was observed and the stimulus was immediately stopped. The response was scored as present or absent after 4 and 16 weeks follow-up time.

Muscle hypertonia: The presence of muscle hypertonia was defined as a spontaneous (stimulus-independent) increase in muscle tone. A positive response was assigned when the partially or fully paralyzed extremity was extended or flexed in the knee or hip joint, and returned to its original position.

### Magnet resonance imaging analysis

To determine the injury dimension, two MRIs were performed at a Siemens Magnetom Avanto 1.5 T (Siemens Romania, Bucharest, Romania). The first complete spine MRI (T1-weighted and T2-weighted scans) was done 6 h after SCI according to standard acquisition procedures, and the second complete spine MRI (T1-weighted and T2-weighted scans) was performed 4 weeks after SCI. The corresponding Digital Imaging and Communications in Medicine (DICOM) files were correlated to each other.

### Tissue processing for immunohistochemistry

At the end of the follow-up period, the minipigs were sacrificed by an overdose intracardial barbiturate injection in deep general anesthesia. The spinal cord tissue was taken at level Th9 to L1, single segments of the rostral (Th9-Th10), lesion site (Th11-Th12), and caudal (Th13-L1) segments prepared and immersion-fixed in 4% paraformaldehyde in 0.1 M phosphate-buffered saline (PBS) at 4 °C for 12 h. Thereafter, the specimens were extensively washed in 0.1 M PBS and stored in 0.1 M PBS with 0.05% sodium azide (NaN_3_) at 4 °C until cryo-sectioning. For cryo-sectioning, the segments were incubated in 15% sucrose in 0.1 M PBS for 24 h and in 30% sucrose in 0.1 PBS for 48 h. Thereafter, the segments were cryofrozen via Tissue Tek OCT™ compound (Hartenstein, Germany). Transverse serial sections (10 μm) were obtained in a Leica cryostat, collected on Superfrost Plus slides, and stored at − 20 °C until further processing. Slides were thawed, washed with 0.1 M PBS with 0.1% Tween 20 (PBST), and blocked with 0.1% bovine serum albumin, 0.1% Triton in PBST (blocking buffer) for 2 h at room temperature. Primary antibodies (anti-200-kD neurofilament heavy (ab8135, Abcam, Cambridge, UK), anti-NeuN (clone A60, Merck Millipore, Darmstadt, Germany), anti-Iba1 (ab5076, abcam), anti-GFAP (ab4648, abcam), and anti-Olig-2 (ab109186, abcam) diluted in blocking buffer were incubated for 24 h at 4 °C. After several washes with PBST, sections were incubated with species-specific Alexa™ fluorochrome-labeled secondary antibodies for 4 h at room temperature. After several washes with PBST and PBS, sections were mounted with an anti-fade mounting medium (Thermo Fischer Scientific, MA, USA) and representative images of each segment were obtained using an Olympus VS 120 (Olympus Corporation, Tokyo, Japan) and post-processed with the OlyVIA Software (Olympus Corporation, Tokyo, Japan) and ImageJ (open source program, developed by the National Institutes of Health, USA).

## Results

### Feasibility of image-guided minimal invasive spinal cord injury in minipigs

**Fig. 1:**
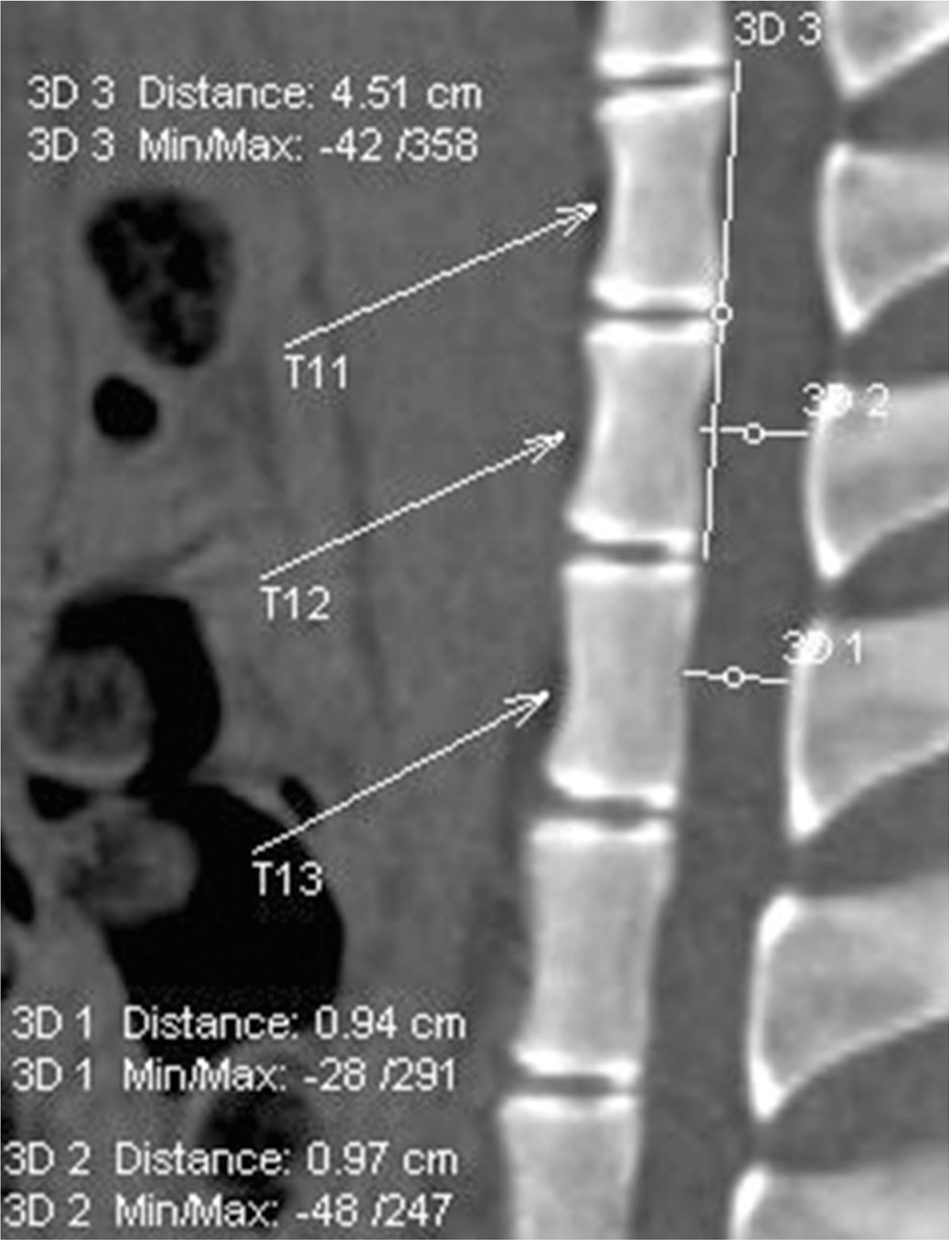
Initial computed tomography (CT) scan with measurements of the dimensions of the spinal cord length and diameter of the area of interest later for spinal cord balloon compression. The length of the spinal cord from thoracic (T) spinal cord level T11 to T13 is 4.51 cm. The diameter at T12 is 0.97 cm and at T13 0.94 cm

**Fig. 2:**
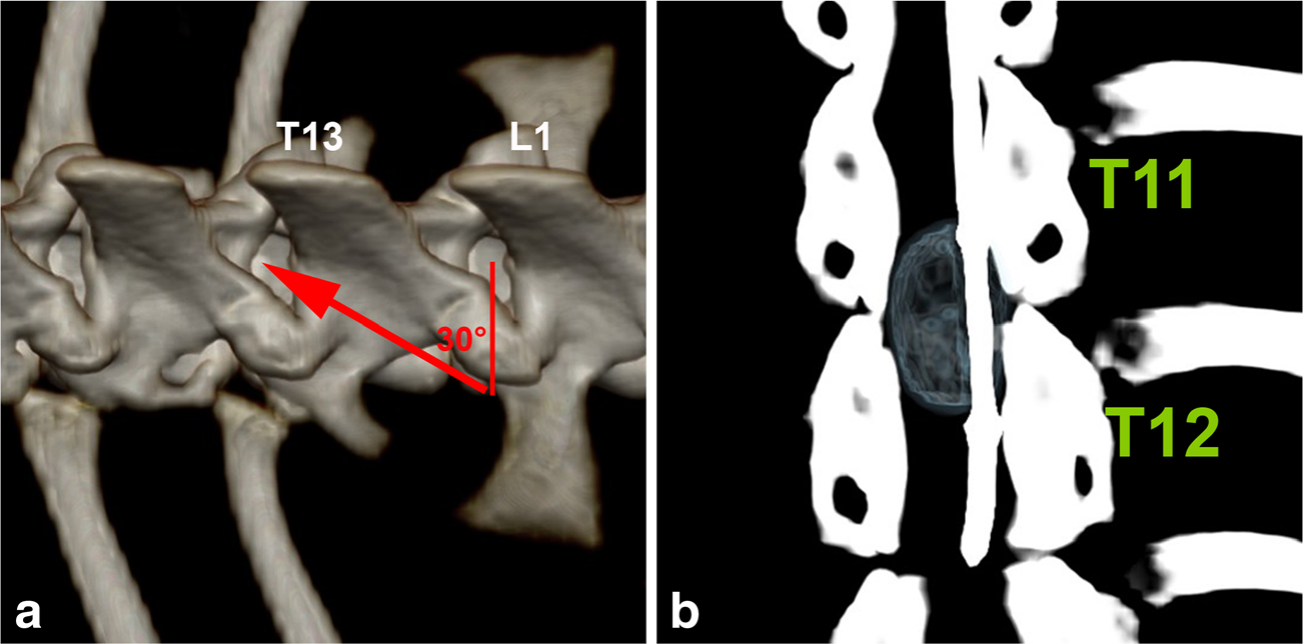
Angle calculation for needle puncture and balloon compression. **a** Needle angle for puncture of the intervertebral space T13/L1. The depicted needle angle is 30°, calculated by a dash to the intervertebral space L1/L2. **b** Inflated balloon during the spinal cord compression phase at thoracic spinal cord level T12/T13

In minipigs, an extradural spinal cord compression was performed by inserting a balloon catheter via the Seldinger technique at the thoracic spinal cord level T12. Inflating the balloon to 2 atm for 30 min led to a complete SCI. All procedures were CT-controlled and images taken for verification of technique. The initial CT scan showed no skeletal abnormalities or deformations in any of our minipigs which could have hampered a needle puncture after Seldinger technique and insertion of the kyphoplasty catheter in any of the ten minipigs. Moreover, it depicted similar thoracic spinal cord dimensions of the ten minipigs with the length of the spinal cord from T11 to T13 being 4.51 ± 0.08 cm and the diameter of the spinal cord being 0.97 ± 0.03 cm at T12 and 0.94 ± 0.02 cm at T13 (Fig. 1). A needle angle of 25–30°, depending on the individual anatomical features, was chosen to correctly and safely puncture the intervertebral space (Fig. 2a). The test to aspirate cerebrospinal fluid (CSF) was negative in all minipigs and the tip of the needle was not placed deeper than the epidural fatty tissue. The hydrophilic guidewire was smoothly rooted up to Th8. Only twice, the guidewire had to be corrected after initial caudal placement by retraction and rerouting to cranial Th8. Dilatation of the intervertebral space with both 10- and 12-French dilator was feasible and possible from an anatomical point of view. Careful dilatation was necessary to avoid spinal cord damage and damage of the dilator which would hamper a safe removal of the dilator. The dimensions of the kyphoplasty catheter fitted to the bony structures of the spinal channel (Fig. 2b). However, any balloon catheter had to be measured in advance to match the dimension of the spinal cord. The balloon with air filling at 2 atm was possible in all minipigs, and no losses were registered during the 30-min compression time. The insertion procedure and placement was easily possible in all ten minipigs. Altogether, the SCI procedure was easily standardized. The procedure was performed exactly the same in all ten minipigs. The procedure took approximately 20 to 30 min for correct epidural balloon catheter placement plus 30 min for the complete compression of the spinal cord.

All ten minipigs survived the spinal cord compression procedure without any complications. No swelling or inflammation was observed above the injury site during follow-up by the regular health checkups. After 5 days post injury, no further antibiotic or analgesic coverage was needed. Body balance, to get in an upright position and to move around the pen by using front limbs solely, was quickly regained after SCI.

### Complete balloon compression-mediated spinal cord injury produces severe neurological deficits in minipigs

All minipigs showed stable severe neurological deficits (grade 0 in the PNM score) in the hind limbs which remained unchanged at follow-up time points of 4 and 16 weeks post SCI. All animals showed a baseline increase in muscle tone (i.e., spastic hypertonia) in the hind limbs during the 4 months follow-up time. Moreover, spastic tail movements were observed starting after 4 weeks of SCI in all SCI minipigs.

Analysis of withdrawal response to a mechanical stimulus for sensational analysis showed a normal response for the front limbs, while the response of the hind limbs was absent during the 4 months follow-up time in all minipigs.

### Balloon compression produces long-lasting complete spinal cord injury in minipigs

**Fig. 3:**
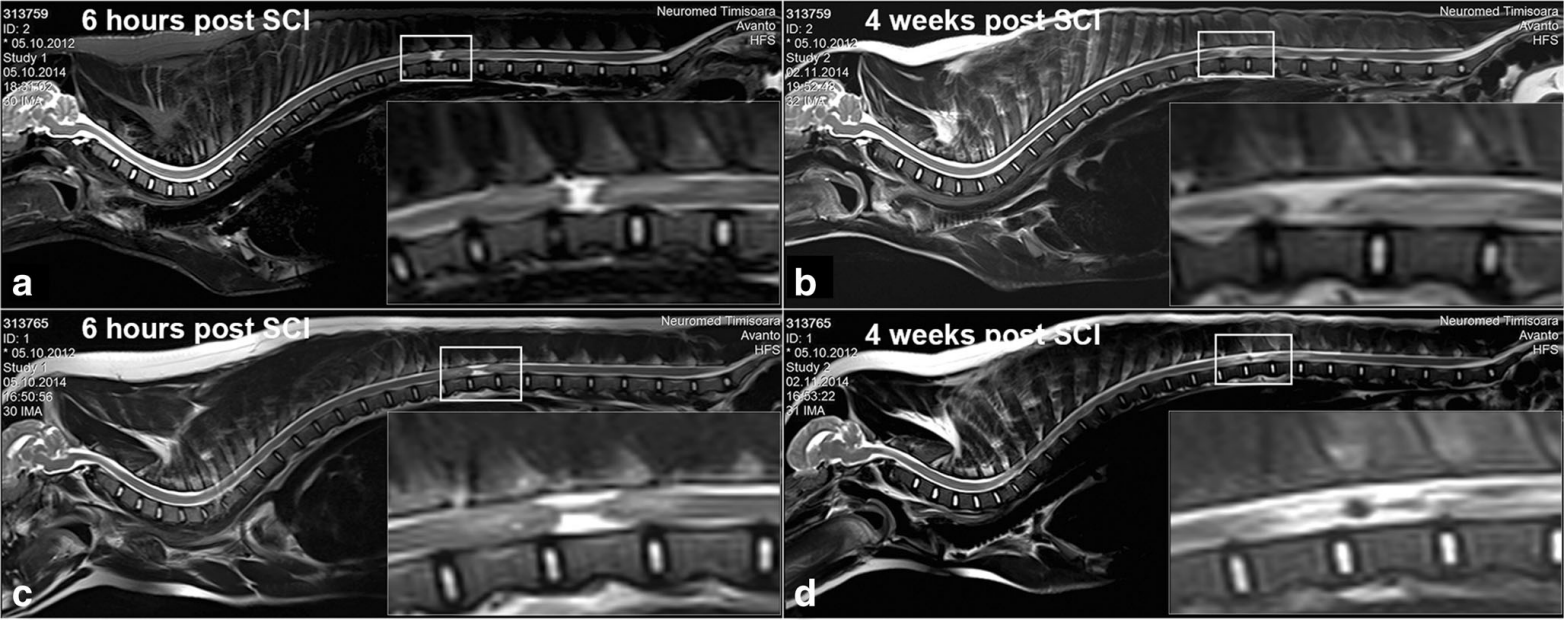
Magnetic resonance imaging (MRI) of the spinal cord of spinal cord injury (SCI) minipigs. **a**, **c** Complete spine MRI’s 6-h post SCI. The selected rectangle shows the lesioned region in higher magnification (lower right corner). **b**, **d** Complete spine MRI’s 4 weeks post SCI. The selected rectangle shows the lesioned region in higher magnification (lower right corner)

To evaluate the extent of the initial damage and to visualize later alterations in the lesion size/dimension, MRI image analysis was performed. The complete spine MRI image assessment at the sub-acute time point of 6-h post injury and the early chronic time point at 4 weeks post injury suggested that (i) the injury was rather complete and (ii) that the injury site remained clearly discernible with comparable lesion size at the two time points (Fig. 3a–d).

When dissecting the spinal cord, the epidural sack was not lesioned due to the balloon catheter compression in any of the ten SCI minipigs. Only after opening the epidural sack, the macroscopic alterations were seen at the spinal cord level of balloon catheter compression.

**Fig. 4:**
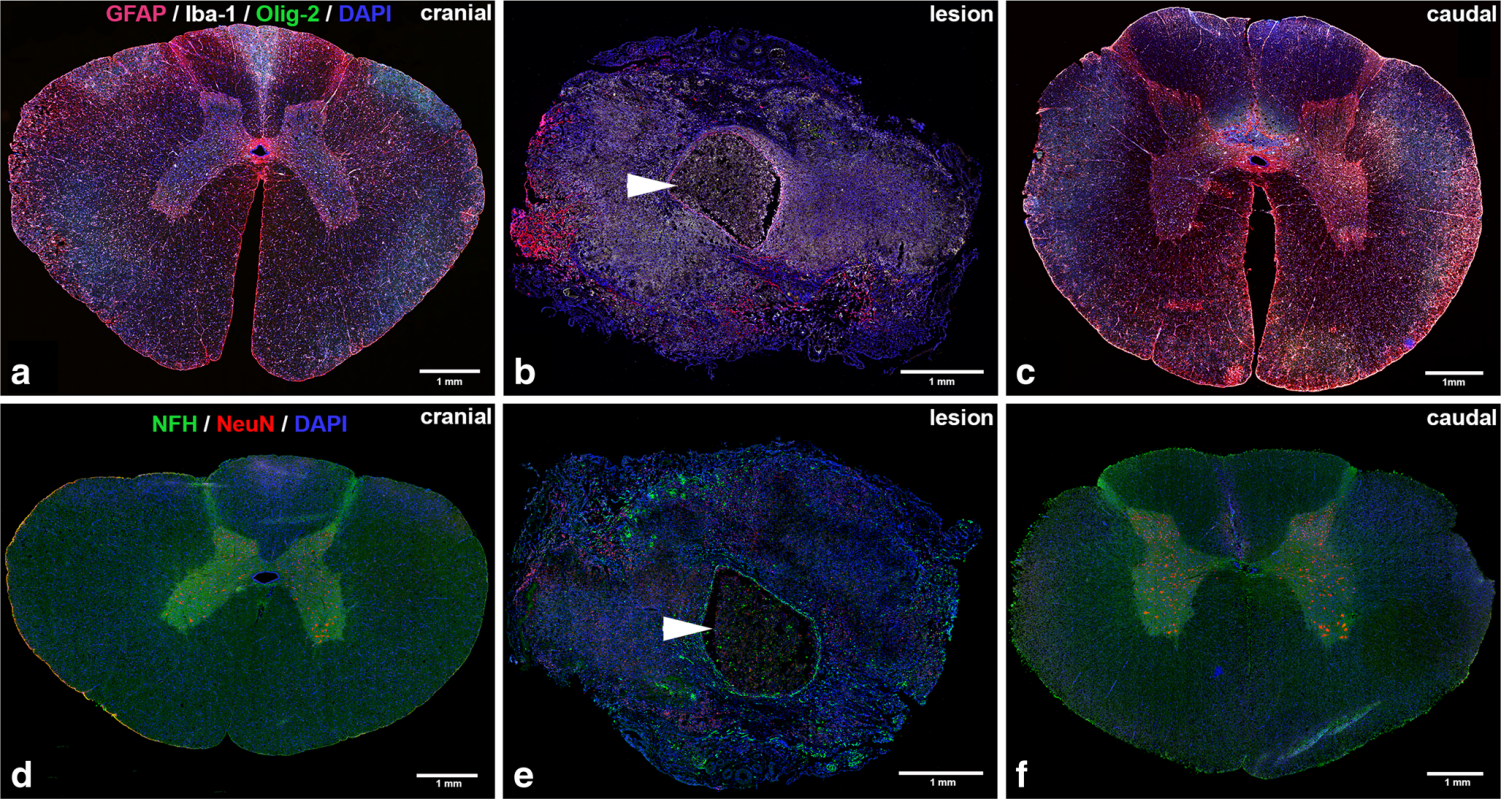
Immunohistochemical stainings of cross sections of the spinal cord of spinal cord injury (SCI) minipigs. **a**–**c** Cross sections of SCI minipig spinal cord tissue rostral to the lesion (thoracic spinal cord level T8), at the lesion site (thoracic spinal cord level T12) and caudal to the lesion (lumbar spinal cord level L2) stained for astrocytes (GFAP, red), microglia (Iba-1, white), oligodendrocytes (Olig-2, green), and nuclei (DAPI, blue). **d**–**f** Cross sections of SCI minipig spinal cord tissue rostral to the lesion (T8), at the lesion site (T12) and caudal to the lesion (L2) stained for neurons (NeuN, red), axons (NFH, green), and nuclei (DAPI, blue). Arrowheads indicate the central cystic cavity. All shown scale bars are 1 mm

The immunohistochemical analysis clearly depicted a normal appearing structural organization of the spinal cord tissue rostal and caudal to the lesion site (Fig. 4a, c, d, f). The central canal was clearly discernible rostral to the lesion site and continued to be seen after the end of the lesion zone at level T13/L1 towards caudal. Neuronal soma were present in the gray matter and their axons appeared dense and well organized in the white matter. Oligodendrocytes, astrocytes, and microglia appeared normally distributed and shaped.

Within the lesion site, a massive disorganization was observed with the presence of cystic cavities in the central gray matter lined by reactive astrocytes (Fig. 4b). The central canal was not to be defined within the lesion site. Furthermore, neuronal cell death was observed by a complete loss of neuronal soma signal and only unorganized fragments of axons present at all (Fig. 4e). The diameter of the spinal cord at the lesion region was approximately 50% smaller compared to the same thoracic region of the healthy reference minipig spinal cords.

## Discussion

This new CT-guided balloon catheter-mediated SCI technique for minipigs represents a minimal invasive alternative approach to the existing invasive techniques. Disadvantages of the standard invasive methods in comparison to the here-presented minimal invasive method are potential blood loss, epidural vein rupture, CSF leakage, cyst formation, and slow recovery from a medium-large open surgery in an animal, which has been already weakened by the onset of the spinal cord trauma. In addition, more invasive methods require a high amount of surgical preparation [6] and longer anesthesia time, which in the case of (mini)pigs is relevant as they are sensitive to long anesthesia regimes and may take longer to recover. Moreover, typical experimental invasive SCI methods require laminectomy, which may influence recovery and regeneration by amplifying inflammation in the spinal cord, which ultimately might mask the effects of applied therapies. Also, laminectomy in quadrupeds leads to a destabilization of the spinal column causing altered body core balance and pain, and may also hamper follow-up monitoring and strike the results of a study. Another issue that typically occurs in invasive SCI methods is the severance of back skeletal muscles to approach the spinal processes, which may also lead to changes in posture, induce extended inflammation, and cause pain. The present approach completely avoids these issues, as no laminectomy is required and back skeletal muscles are only minor disrupted during the needle puncture. So far, only one SCI method by means of an epidural balloon compression is described in the literature, in dogs [16, 17]. In contrast to the present work, neither needle placement nor spinal cord compression was image controlled. Therefore, correct placement or balloon catheter function was demonstrated in these studies.

Depending on the purpose of the study and on the underlying question, different tSCI regimes are available for (mini)pigs to choose upon as for example the weight drop or the pneumatic impactor technique [13]. These procedures may spare nerve fibers and are therefore models of incomplete SCI, which may not adequately address key questions of a study design. The described new CT-guided balloon compression injury procedure excludes the sparing of nerve fibers. The inflated balloon completely filled the spinal channel and bluntly transected the spinal cord using air pressure and applied time. This was shown intraoperatively by CT images, proven by MRI images at 6 h and 4 weeks after injury and finally verified by immunohistochemical analyses of the dissected spinal cord tissue after the end of the 4-month follow-up time. Moreover, the stable neurological deficits shown during the 4-month follow-up time were in compliance with the MRI and immunohistochemical results and demonstrated complete SCI. However, similar to the forceps compression model, balloon compression typically lacks the highly acute component of tSCI [6]. However, a rapid inflation of the balloon can add aspects of this type of trauma, which is technically easy to achieve and monitor during compression time. Furthermore, the CT-controlled approach guarantees a 100% standardized procedure that can be continuously monitored. As known from the dog model, a 10-min compression time leads to complete paraplegia [16]. However, due to the larger diameter of the porcine spinal cord, a 30-min compression regime was chosen and ruled out incompleteness or surviving nerve fibers. In comparison to the water-filled balloon used in dogs, we chose air to fill the balloon of the catheter. However, there is no difference in the impact of the traumatic event based on the filling substance. More important is the verification of the filling state and to maintain it at 100% during the compression time.

A clear limitation of the (mini)pig as a translational model for human SCI is the fact that (mini)pigs lack fine neuroanatomic/functional organization of the motor system, which is found in human and non-human primates. Thus, the (mini)pig model is not be suitable to detect the discrete therapeutic effects associated with the protection and/or sprouting of limited and functionally distinct populations of motor axons [7].

In summary, the minipig balloon catheter spinal cord injury model represents a minimal invasive alternative large animal model to already existing medium to large animal SCI models such as the dog, cat, and non-human primates. It is characterized by a consistent, predictable, and stable neurological deficit after the balloon compression spinal trauma, as well as by the development of spinal cord histopathology comparable to changes observed in patients [7]. The study demonstrates that this new minimal invasive, CT-guided technique might be an appropriate technique for studying therapeutic approaches that require a complete SCI model and high inter-animal comparability. The performed characterization of the post-injury development and neurological assessment during 4 months of chronic complete SCI have further demonstrated that it is an appropriate in vivo animal model of complete SCI to answer questions regarding pathological changes, therapeutic safety, and treatment efficacy, especially when a close translation to humans and human physiology is important.

## References

[1] Ahuja CS, Wilson JR, Nori S, Kotter MRN, Druschel C, Curt A, Fehlings MG (2017). Traumatic spinal cord injury. Nat Rev Dis Prim.

[2] Munce SE, Perrier L, Tricco AC, Straus SE, Fehlings MG, Kastner M, Jang E, Webster F, Jaglal SB (2013). Impact of quality improvement strategies on the quality of life and well-being of individuals with spinal cord injury: a systematic review protocol. System Rev.

[3] Kornhaber R, McLean L, Betihavas V, Cleary M (2018) Resilience and the rehabilitation of adult spinal cord injury survivors: a qualitative systematic review. J Adv Nursing 74(1):23–33. 10.1111/jan.13396 10.1111/jan.13396 28726274

[4] Ahuja CS, Nori S, Tetreault L, Wilson J, Kwon B, Harrop J, Choi D, Fehlings MG (2017). Traumatic spinal cord injury—repair and regeneration. Neurosurgery.

[5] Dietrich WD (2003). Confirming an experimental therapy prior to transfer to humans: what is the ideal?. J Rehabil Res Dev.

[6] Cheriyan T, Ryan DJ, Weinreb JH, Cheriyan J, Paul JC, Lafage V, Kirsch T, Errico TJ (2014). Spinal cord injury models: a review. Spinal Cord.

[7] Navarro R, Juhas S, Keshavarzi S, Juhasova J, Motlik J, Johe K, Marsala S, Scadeng M, Lazar P, Tomori Z, Schulteis G, Beattie M, Ciacci JD, Marsala M (2012). Chronic spinal compression model in minipigs: a systematic behavioral, qualitative, and quantitative neuropathological study. J Neurotrauma.

[8] Sharif-Alhoseini M, Khormali M, Rezaei M, Safdarian M, Hajighadery A, Khalatbari MM, Safdarian M, Meknatkhah S, Rezvan M, Chalangari M, Derakhshan P, Rahimi-Movaghar V (2017). Animal models of spinal cord injury: a systematic review. Spinal Cord.

[9] Leonard AV, Menendez JY, Pat BM, Hadley MN, Floyd CL (2017). Localization of the corticospinal tract within the porcine spinal cord: implications for experimental modeling of traumatic spinal cord injury. Neurosci Lett.

[10] Dalmose AL, Bjarkam CR, Djurhuus JC (2005). Stereotactic electrical stimulation of the pontine micturition centre in the pig. BJU Int.

[11] Jensen KN, Deding D, Sorensen JC, Bjarkam CR (2009). Long-term implantation of deep brain stimulation electrodes in the pontine micturition centre of the Gottingen minipig. Acta Neurochir.

[12] Mills IW, Drake MJ, Greenland JE, Noble JG, Brading AF (2000). The contribution of cholinergic detrusor excitation in a pig model of bladder hypocompliance. BJU Int.

[13] Jones CF, Lee JH, Kwon BK, Cripton PA (2012). Development of a large-animal model to measure dynamic cerebrospinal fluid pressure during spinal cord injury: laboratory investigation. J Neurosurg Spine.

[14] Lee JH, Jones CF, Okon EB, Anderson L, Tigchelaar S, Kooner P, Godbey T, Chua B, Gray G, Hildebrandt R, Cripton P, Tetzlaff W, Kwon BK (2013). A novel porcine model of traumatic thoracic spinal cord injury. J Neurotrauma.

[15] McInnes EF, McKeag S (2016). A brief review of infrequent spontaneous findings, peculiar anatomical microscopic features, and potential artifacts in Gottingen minipigs. Toxicol Pathol.

[16] Fukuda S, Nakamura T, Kishigami Y, Endo K, Azuma T, Fujikawa T, Tsutsumi S, Shimizu Y (2005). New canine spinal cord injury model free from laminectomy. Brain Res Brain Res Protocol.

[17] Tarlov IM, Klinger H (1954). Spinal cord compression studies. II. Time limits for recovery after acute compression in dogs. AMA Arch Neurol Psychiatr.

